# Comparative study of minutiae selection methods for digital fingerprints

**DOI:** 10.3389/fdata.2023.1146034

**Published:** 2023-04-18

**Authors:** Benoit Vibert, Jean-Marie Le Bars, Christophe Charrier, Christophe Rosenberger

**Affiliations:** Normandie Univ, UNICAEN, ENSICAEN, CNRS, GREYC, Caen, France

**Keywords:** fingerprint, minutiae selection, matching algorithm, template reduction, benchmarking

## Abstract

Biometric systems are more and more used for many applications (physical access control, e-payment, etc.). Digital fingerprint is an interesting biometric modality as it can easily be used for embedded systems (smartcard, smartphone, and smartwatch). A fingerprint template is composed of a set of minutiae used for their comparison. In embedded systems, a secure element is in general used to store and compare fingerprint templates to meet security and privacy requirements. Nevertheless, it is necessary to select a subset of minutiae from a template due to storage and computation constraints. In this study, we present, a comparative study of the main minutiae selection methods from the literature. The considered methods require no further information like the raw image. Experimental results show their relative performance when using different matching algorithms and datasets. We identified that some methods can be used within different contexts (enrollment or verification) with minimal degradation of performance.

## 1. Introduction

In our daily lives, we increasingly use smart objects such as smartphones, smartwatches, smart cards, etc., as a physical gateway to our digital services. To meet security trends, biometric is often applied for user authentication (Liu et al., 2022; Singh and Kant, 2022) to replace passwords. Digital fingerprint is a well-known morphological biometric modality (Maltoni et al., 2009) with many advantages for such embedded devices. A fingerprint capture is fast and convenient for the user. A fingerprint sensor can also be embedded in smart objects such as a smartwatch. In terms of processing, a fingerprint template is represented by a low-size feature vector (set of minutiae), and the comparison is very fast (<500*ms*). A biometric system includes two steps, such as 1) enrollment and 2) verification. A matching algorithm computes a comparison score between a probe biometric template and the reference one. A fingerprint template is, in general, composed of a set of specific points called minutiae *m*_*i*_, 1 ≤ *i* ≤ *N* (*N* is the number of minutiae in the template). A minutia is usually described by four values *m*_*i*_ = (*x*_*i*_, *y*_*i*_, *T*_*i*_, and θ_*i*_), where (*x*_*i*_, *y*_*i*_) is the location of the minutiae in the image, *T*_*i*_ is its type (bifurcation, ridge ending, etc.), and θ_*i*_ is its orientation (related to the ridge).

Modern biometric systems consider security and privacy by design. As a common practice, a fingerprint template is stored in a secure element (SE), following the ISO Compact Card standard (ISO, b) to ensure the interoperability between biometric sensors and systems (Grother and Salamon, 2007). An SE has hardware and software constraints such as the size of memory and the number of data we can send with an APDU (Application Protocol Data Unit) command (ISO, 1987). These limitations have an impact on the embedded system and the size of the fingerprint template. The ISO/IEC 19794 − 2 standard recommends that the maximal number of minutiae for biometric reference and probe ISO-CC templates is 60 (ISO, 2011). However, in an operational biometric system, a fingerprint template is usually limited to a specific number of minutiae which is lower or equal to 50 to satisfy the memory space, the APDU specifications, and also the verification time. In this case, it is necessary to reduce the template size when the extractor has detected more minutiae. The aim of this study is to study and determine the best algorithmic solutions to select these minutiae without any *a priori* information (no access to the fingerprint image or minutiae quality scores which prevents to delete the more suspicious minutiae). An optimal template reduction method should be able to limit the decrease in performance when using less minutiae. Testing all combinations (selection of a subset of minutiae) is not possible for computation limitations. Few algorithms for minutiae selection have been proposed in the literature (ISO, 2007; Vibert et al., 2015, 2018), and the scope of the proposed study is to compare the main methods.

The contributions of this study are as follows:

- Identification of main methods in the literature for the minutiae selection from digital fingerprint templates.
- Study of the performance (degradation of recognition) and efficiency (computation time) when selecting a subset of minutiae from the initial template.
- Use of three fingerprint datasets from different sensors/resolutions and three matching algorithms to draw general conclusions.
- Identification of the context of the use of each tested method (enrollment/verification) for an operational application.

This study is organized as follows: Section 2 recalls the existing methods of the literature for template reduction; Section 3 defines all the components of the experimental protocol we followed; Experimental results are exposed in section 4; Section 5 concludes this study and gives some perspectives.

## 2. State-of-the-art methods

This section is devoted to an optimization problem. Given a fingerprint template containing *N* minutiae, we wish to determine the optimally reduced template composed of *N*_*max*_ < *N* minutiae. For this purpose, we suppose that we have a matching algorithm returning a score, and we assume that the best-reduced template is the one that maximizes the score with the initial template (with all extracted minutiae). Under this assumption, to determine this optimal template, we should test $\left(\begin{array}{c} N_{max}\\ n \end{array}\right)$ possibilities (number of combinations of *N*_*max*_ among *N*). However, it is not feasible to test all the possibilities, for instance, with *N* = 50 and *N*_*max*_ = 30, there are 4.7*10^13^ possible reduced templates. This is why we need an optimization method that is significantly less costly. Many methods such as simulated annealing or tabu method are available.

Previous studies suggest that the most relevant minutiae are close to the core point (point on ridges with the maximal curvature value), and therefore, most proposed solutions in the literature are based on this assumption (Julasayvake and Choomchuay, 2007; Khodadoust and Khodadoust, 2017; Sharma and Dey, 2019; Win et al., 2019). Our results contradict this hypothesis. First of all, in this study, we propose, a ground truth solution to enlighten the performance we can hope to reach. In addition, this solution aims to provide a good idea of the spatial distribution of the selected minutiae. It exploits the score of a matching algorithm by building a template reduction that maximizes the matching score between the original template (with all the minutiae) and the reduced one. This approach may not be applied in an SE (mainly for computation time reason). Indeed, the matching algorithm used in the SE does not return a score but just a Boolean decision value for evident security reasons. It seems unlikely to derive an algorithm with a non-prohibitory cost due to the combinatorial explosion of the number of possible reduced templates. Second, we study methods without any auxiliary knowledge forcing us to exploit only the spatial distribution of the minutiae.

We present, in this section, the main methods for template reduction of digital fingerprints in the literature.

### 2.1. Truncation

This simple method, defined by the ISO 19795-2 (ISO, 2007), is based on a simple truncation, only the first *N*_*max*_ minutiae of the initial template are kept. The reason why this simple approach could be efficient is related to the generation of the initial fingerprint template. For some commercial biometric systems, a fingerprint template is generated with the ascending order of the minutiae on the Y axes. In the case of some captures have been made, minutiae with high quality (always present on the different captures, for example) could be placed at the beginning of the minutiae template. Then, the selection of the *N*_*max*_ first minutiae could be very simple and efficient. Algorithm 1 shows the different steps to obtain the reduced template with this method.

**Algorithm 1:**
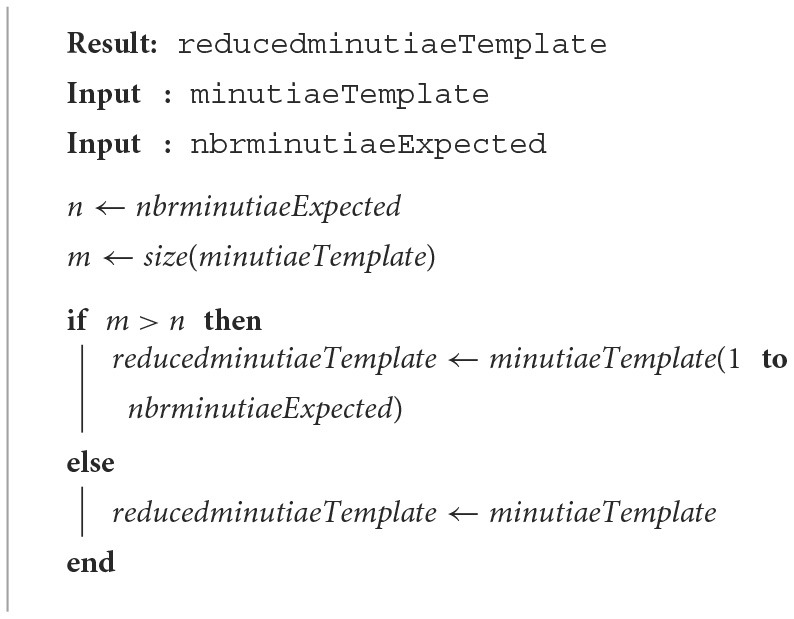
Truncation method.

### 2.2. Random truncation

This method, named RandTrunc, is based on the random permutation of the initial template where the *N*_*max*_ first minutiae are kept. *N*_*max*_ represents the number of desired minutiae for the final reduced template. We assume each minutia has the same probability to be selected and follows a standard normal distribution. This method allows to test whether all the minutiae may be useful in the matching algorithm too. The randPerm(m) function performs this permutation, and the proposed scheme is presented in Algorithm 2.

**Algorithm 2:**
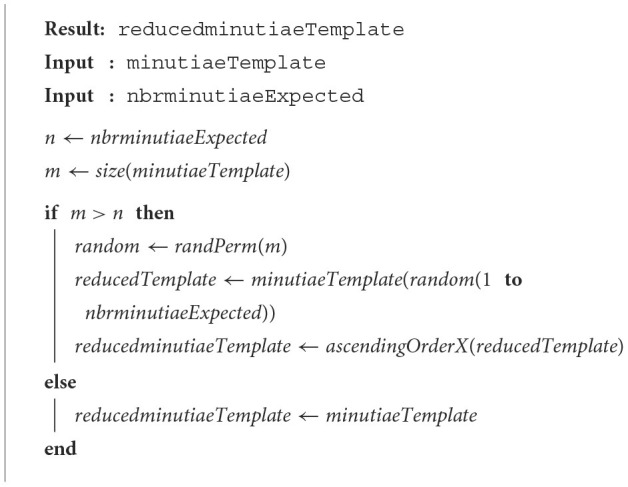
Random truncation method.

### 2.3. Barycenter

This method is based on the peeling mechanism, and it is fast and simple (a computation of a few milliseconds). The NIST has observed that minutiae nearest the core point of the fingerprint are the most involved in the comparison process (Grother and Salamon, 2007). In our context, we cannot compute precisely the CORE point (the fingerprint image is not known); nevertheless, the centroid of the minutiae template is often a good estimate. With this approach, we only keep the closest minutiae to the centroid. Algorithm 3 contains the required instructions.

**Algorithm 3:**
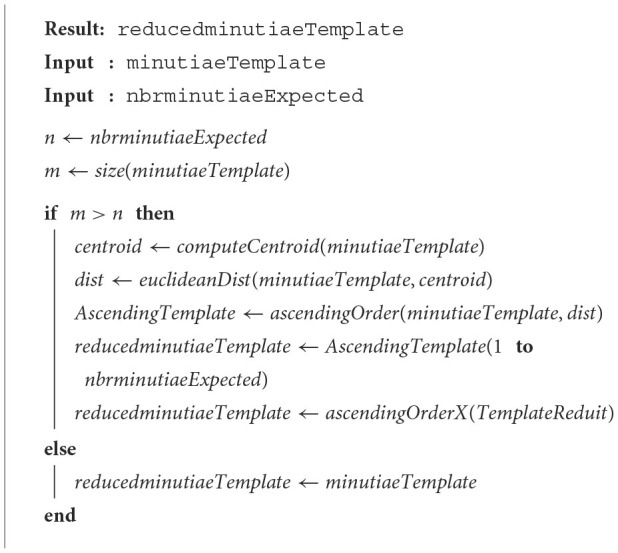
Barycenter method.

The ascendingOrder function orders the minutiae in the function of the distance *d*_*i*_, *i* = 1:*N* with an ascending order. The last method ascendingOrderX orders the minutiae of the template in the function of the *X* element, and this step is necessary since an ISO Compact Card II is composed of this format. Notably, this method will be also useful for the other methods developed and presented further.

### 2.4. Median Y

This method, named Median Y, has the same workflow as the barycenter one, but we only exploit the Y elements, information of the minutiae template. Algorithm 4 presents the developed technique. The computeMedian function refers to the median value Median(*Y*_*m*_) of the template on the Y feature of the initial template. The euclideanDistY function computes the euclidean distance *d*_*i*_(*Y*_*i*_, *Y*_*m*_) between minutiae *Y*_*i*_ of the template and the median value *Y*_*m*_.

**Algorithm 4:**
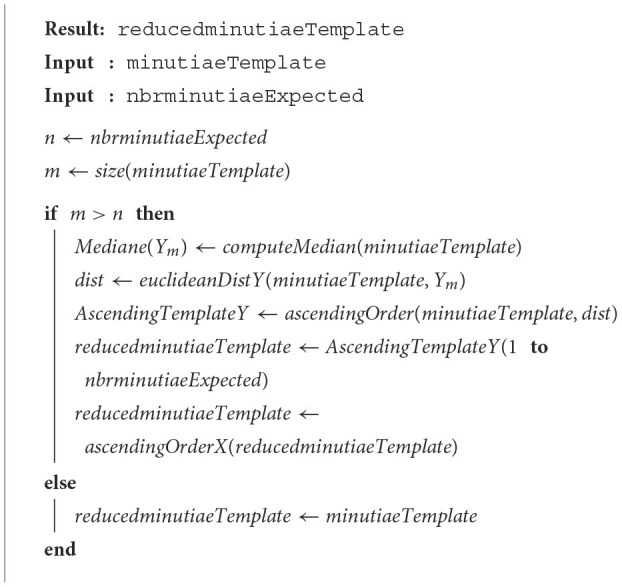
Median Y method.

### 2.5. K-means-based scheme

When assuming the minutiae follow a standard uniform distribution, this does not mean that the same number of selected minutiae are present in each part of the template, that is, there is not a spatial standard uniform distribution of minutiae. Thus, decomposing the template into several areas does not provide a guarantee that the selected minutiae are equally spatially separated. If we want to address the spatial distribution of minutiae, we have to consider a classification-based method. The designed method is based on the Fuzzy C-Means (FCM) (Pal and Bezdek, 1995) algorithm, a well-known unsupervised data classifier. In our study, this method takes as input parameters both the template of minutiae and the number of classes that we expect (i.e., the final number of minutiae to reach). As output, we have the focal point of each class from which we seek the closest minutiae of this point. Algorithm 5 presents the three steps of the proposed k-mean-based technique.

**Algorithm 5:**
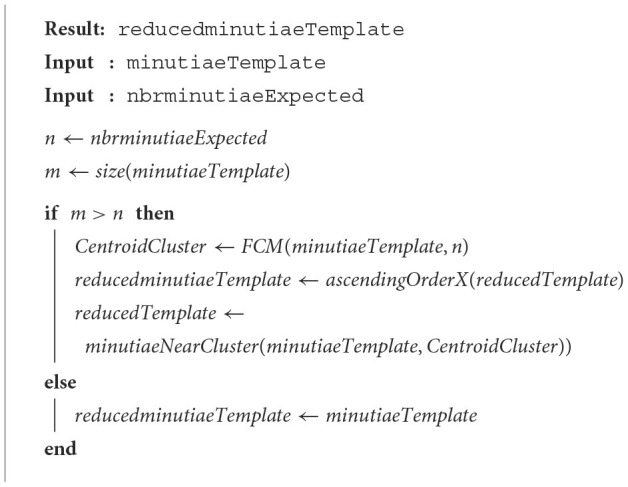
k-means-based method.

In Algorithm 5, the FCM function is divided into two parts: 1) the initialization and 2) the processing phases. They are described as follows:

1. Initialization phase
  (a) Select a random minutia to serve as the centroid *C*_1_ of the first class ℭ_1_.
  (b) Find the minutiae having the greatest distance from the centroid *C*_1_ to serve as the centroid *C*_2_ of the second class ℭ_2_.

2. Processing phase
  (a) Compute distances between each minutia and each class centroid (_*C*_*i*_)2 < *i* < *N*_, where *N* is the number of computed centroid at this step.
  (b) Assign into each class (_ℭ_*i*_)2 < *i* < *N*_ the nearest minutiae to (_*C*_*i*_)2 < *i* < *N*_ in order to generate a Voronoi diagram, where all minutiae of class ℭ_*i*_ are closer to the centroid *C*_*i*_ than to any other. Compute the new value of each centroid (_*C*_*i*_)2 < *i* < *N*_ as the barycenter of each associated class (_ℭ_*i*_)2 < *i* < *N*_.
  (c) Select a new point as a new *C*_*j*_ centroid of the new class (ℭ_*j*_) with a large distance from the other centroids *C*_*i*_. The number of classes is *N*+1.
  (d) Repeat the previous three steps until getting the desired number of classes *N*_*max*_.

The minutiaeNearCluster function returns the closest minutiae to the centroids of the returned classes by the FCM method.

### 2.6. Incremental barycenter

This method is based on the Barycenter method (Section 2.3). The NIST scheme is modified by introducing an incremental method with barycenter. The main idea is to recalculate the barycenter after removing a minutia. Algorithm 6 describes the different steps of the proposed method. The main difference with Algorithm 3 is the deletion of the furthest minutiae from the barycenter. We also repeat the different stages until obtaining the desired number of minutiae.

**Algorithm 6:**
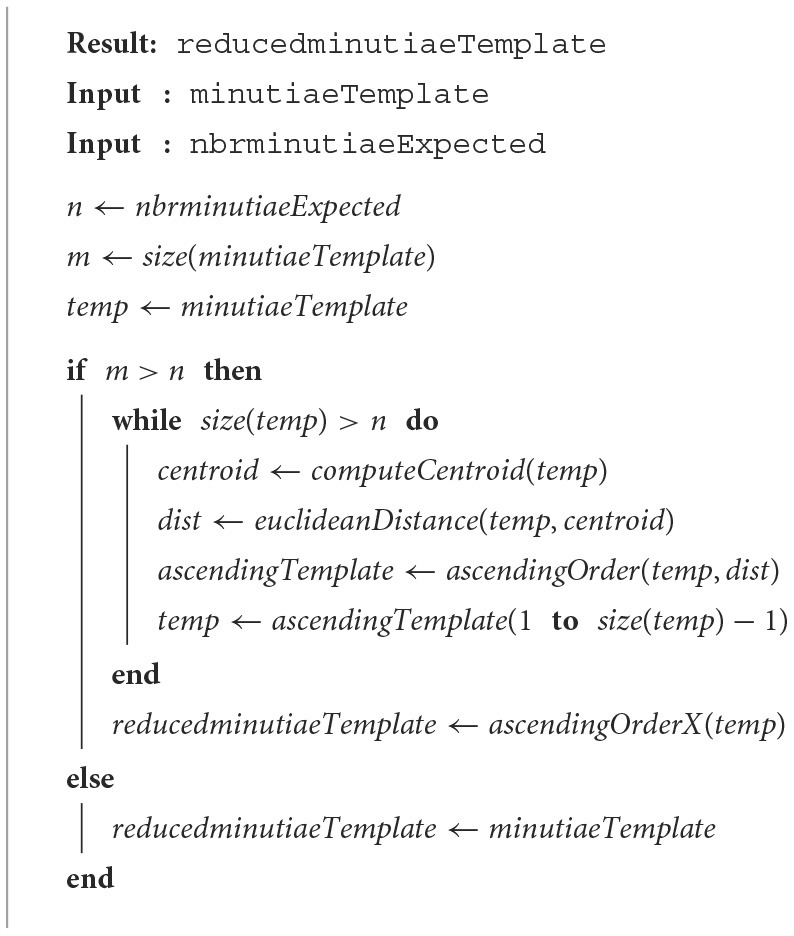
Incremental barycenter.

### 2.7. Minutiae reduction based on a genetic algorithm

In this section, we detail the MRGA method (Minutiae Reduction based on a Genetic Algorithm) to estimate the best possible reduction for a minutiae template (Vibert et al., 2018). A genetic algorithm is used to solve the optimization problem for minutiae selection. Genetic algorithms are adaptive heuristic search algorithms introduced in the 1970s by Holland (1975) and Rudolph (2000). They allow to approximate the optimal value of a criterion by simulating the evolution of a population up to the survival of the best individuals (Wall, 1996). The survivors are obtained by selection, mutation, or crossing of the previous generation. In our context, it is natural to design the evaluation function according to the score of the matching algorithm. A genetic algorithm is defined by five essential elements as follows:

1. **Genotype**: This is a set of characteristics representing each individual in a population. In our case, the initial population consists of 500 individuals composed of *N* elements, *N* is the number of expected minutiae in the reduced biometric template. Since we want to get a template with minutiae present in the initial template, the population will be constituted by random draws of *N* minutiae in the initial template containing *M* minutiae.
2. **Initial population**: This is a set of individuals randomly drawn from the original template. Each individual consists of *N* elements. Each element corresponds to a unique position of a minutia present in the original template.
3. **Evaluation function**: This element measures the quality of an individual *I*_1_ (possible reduced template). We consider the matching score between this individual and its associated initial template (with all minutiae). In this study, we used the MCC comparison algorithm, as it is fast to compare two biometric templates and it has good performance. In conclusion, the higher the similarity score, the better the tested individual.
4. **Operations on genotypes**: The genes of the individual are modified through three functionalities:
  (a) *Selection*: Individuals that do not match the environment (whose score is not sufficient) are not selected. To do this, we select the elite individuals (the five individuals with the highest scores).
  (b) *Crossing*: The genes resulting from the crossing of two individuals are a combination of the genes of their parents. To obtain the individual results from individuals *I*_1_ and *I*_2_, we look at the elements present in the two individuals without the duplicates and randomly select the first *N* elements. We, thus, obtain an individual (son) mixing the genes of the two individuals (parents).
  (c) *Mutation*: Some of the genes are modified in order to better adapt to the environment. We randomly draw an individual, and then, we cross this individual with an elite individual. The resulting individual *I*_*r*_ = mutation and (*I*_1_) = cross(*I*_1_, *I*_*a*_) with the random individual. This enables to obtain an individual having genes from an elite individual and a random one.

5. **Termination**: This is the end-of-evolution criterion depending on the score of individuals or the number of generations. If an individual keeps the same score for 10 generations or 500 generations have been made, the algorithm ends.

We summarize here the work-flow of the execution of a genetic algorithm as follows:

1. Definition of the initial population.
2. Evaluation of individuals.
3. Generation of the following population as follows:
  (a) Selection of five elite individuals;
  (b) 30% of the population (here, 150 individuals) is obtained by mutating elite individuals with random ones.
  3(c) 0% of the population (here, 150 individuals) is obtained by crossing elite individuals.
  (d) Selection of random individuals to complete the population of 500 individuals.

4. Return to step 2 if the stop criterion is not satisfied.

### 2.8. Discussion

**Figure 1:**
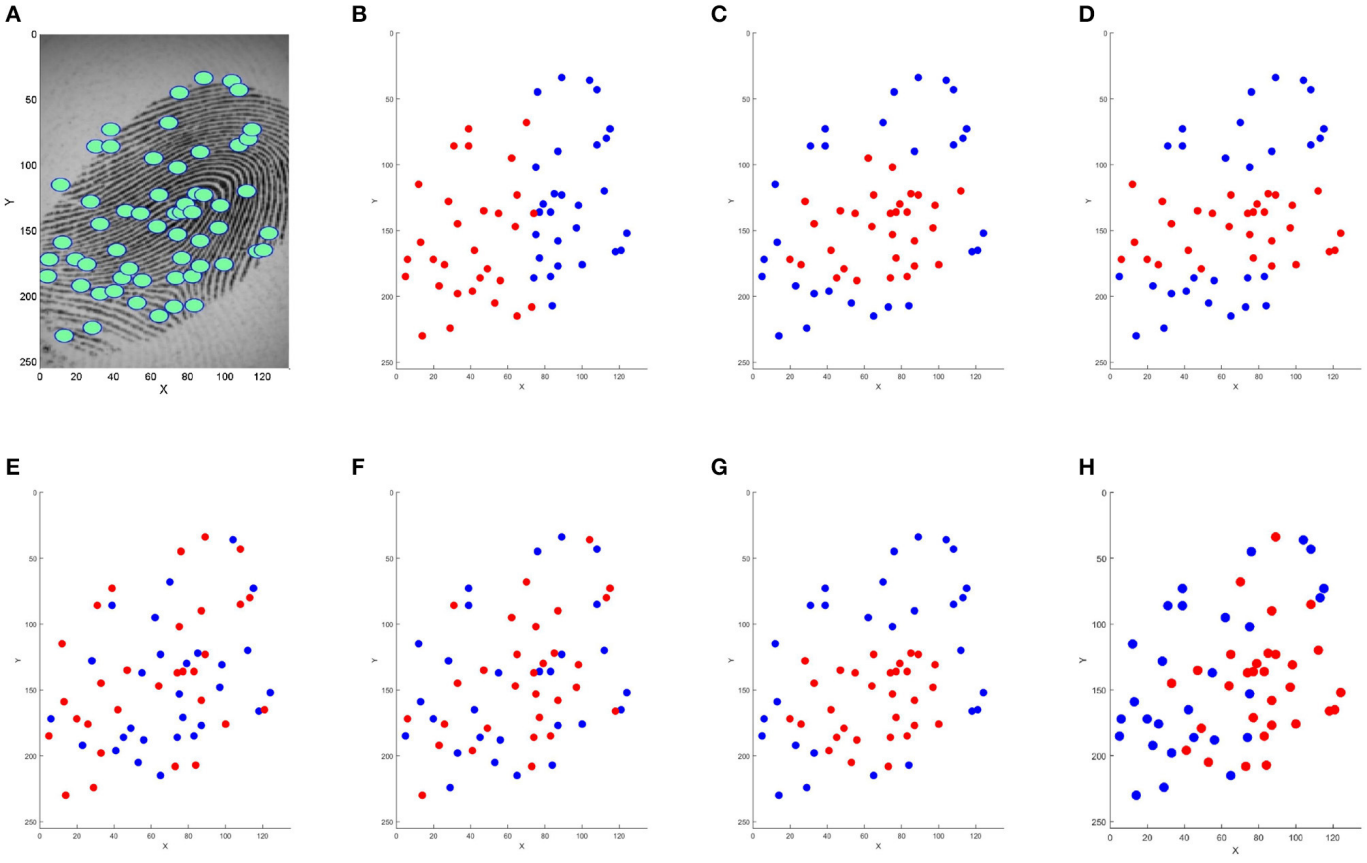
Minutiae selection of the initial template **(A)** with different methods: **(B)** truncation, **(C)** barycenter, **(D)** Median Y, **(E)** truncation random permutation, **(F)** K-means, **(G)** incremental barycenter, and **(H)** MRGA. Minutiae in red are kept on the reduced template with nbrminutiaeExpected = 30.

The presented methods from the literature are very different. Some methods are very simple, such as truncation. Figure 1 shows an illustration of the result of the trial reduction methods on a digital fingerprint by selecting the “best" 30 minutiae (represented in red). It is difficult to quantify their relative efficiency in terms of performance and computation time. Selected minutiae are very different when using all presented methods. The main contribution of this study is to propose a comparative study of these methods in order to answer this question.

## 3. Experimental setup

We define the experimental setup in the following sections.

### 3.1. Databases

In this study, we used three datasets of digital fingerprints composed of 800 images from 100 individuals with eight samples per user. These datasets were used during previous Fingerprint Verification Competitions (FVC):

1. FVC2002 DB2 dataset (Maio et al., 2002): The image resolution is 296 × 560 pixels with an optical sensor “FX2000" by Biometrika.
2. FVC2004 DB1 dataset (Maio et al., 2004): The image resolution is 640 × 480 pixels with a “V300" optical sensor by CrossMatch.
3. FVC2004 DB2 dataset (Maio et al., 2004): The image resolution is 328 × 364 pixels with an optical pickup “U.are.U 4000" by Digital Persona.

**Figure 2:**
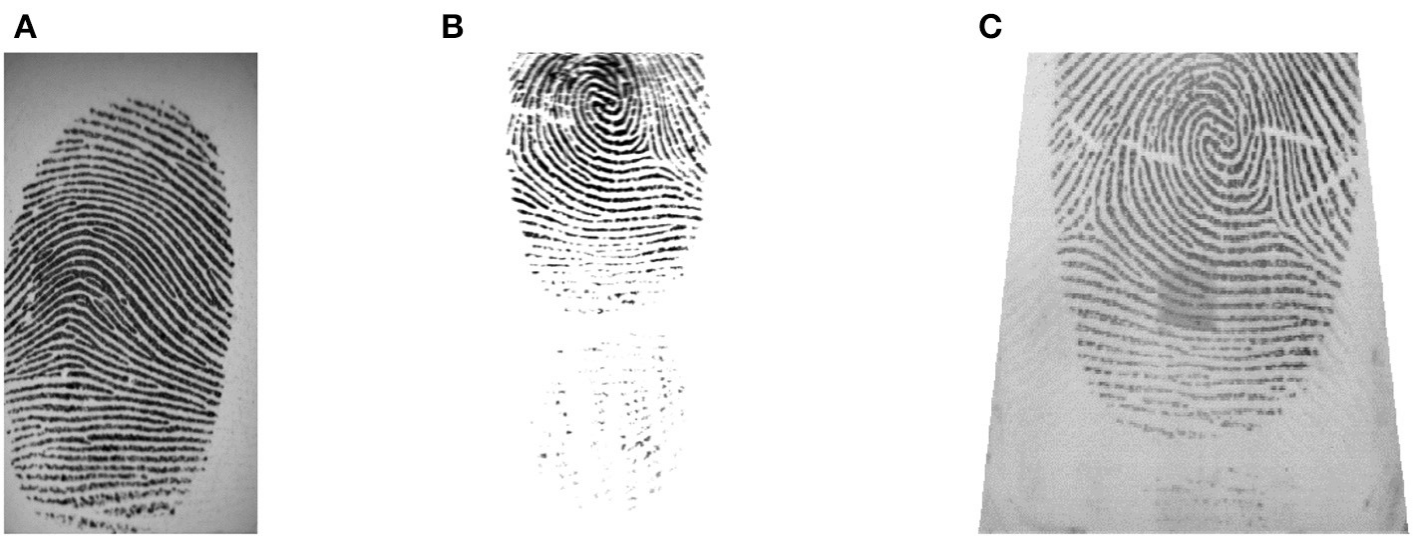
Example of fingerprints from each used database: **(A)** database FVC2002 DB2 **(B)** database FVC2004 DB1, and **(C)** database FVC2004 DB2.

Figure 2 shows a fingerprint sample from each database. This also shows the diversity of fingerprint quality in the considered databases.

**Figure 3:**
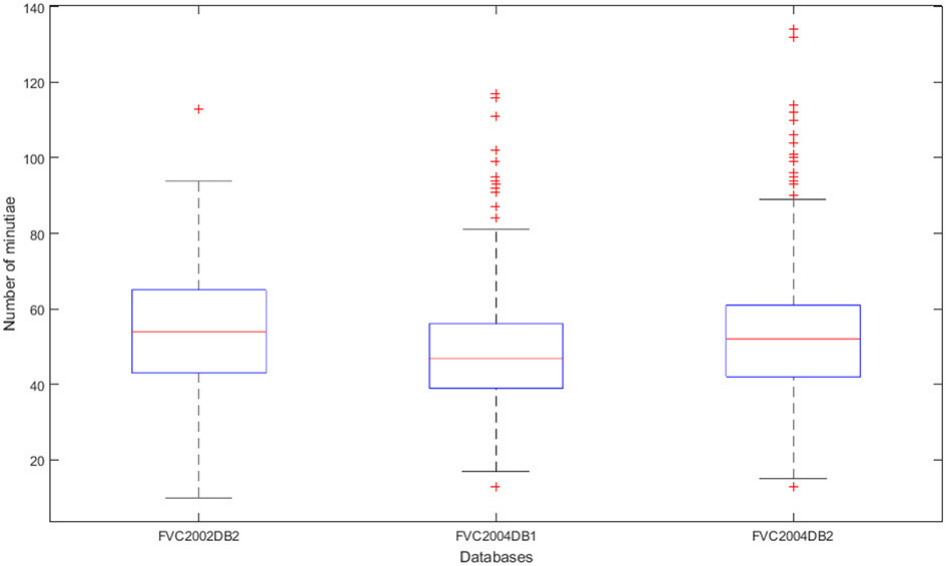
Distribution of the number of minutiae contained in each template per database by box whiskers.

Figure 3 shows the distribution of the number of minutiae for each sample in the biometric datasets. Notably, the number of minutiae is not similar for each dataset, even if they are close on average. The average number of minutiae is 54 for the FVC2002DB2 database, 48 for FVC2004DB1, and 43 for FVC2004DB2. Moreover, the number of minutiae can be greater than 80 or even 100, which is greater than the maximum size accepted by secured elements. This is the reason why we need to reduce the number of minutiae in digital fingerprint templates.

### 3.2. Minutiae extractor

In this study, the minutiae templates are extracted using the NBIS tool, and more specifically MINDTCT (Watson et al., 2007) from NIST. We choose this extractor since it is widely used for academic research.

### 3.3. Matching algorithms

A total of three comparison algorithms are considered to be sure that our conclusions are not dependent on the used matching algorithm. The first two come from the academic world and the last one from the industrial world as follows:

1. **Bozorth3 algorithm** Watson et al. (2007): This matching algorithm takes into account only the locations and orientation of the minutiae to match the fingerprints.
2. **Minutiae Cylinder-Code (MCC) algorithm** Cappelli et al. (2010): The representation of MCC associates a local structure with each minutia. This structure contains the spatial and directional relationships between minutiae and their neighborhood (fixed radius). Each structure is invariant in translation, rotation, distortions, and small errors of extraction of characteristics. A double measure of similarity is calculated and consolidated to provide an overall score for the comparison.
3. **Commercial algorithm**: We do not have any information on how this algorithm works. This commercial matching algorithm is considered a black box, its output is an answer such as “Accepted” or “Declined”, and not a score to avoid hill-climbing attacks (Martinez-Diaz et al., 2006).

### 3.4. Evaluation metrics

**Figure 4:**
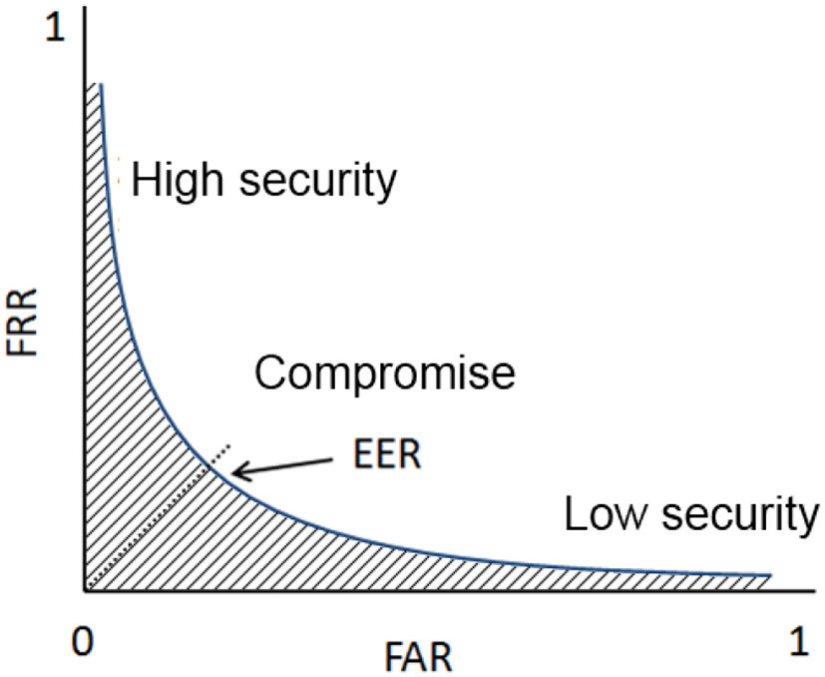
Definition of the ROC curve: evolution of the False acceptance rate vs. the false rejection rate.

To assess the performance of each template reduction method, the first sample of each individual is chosen as a reference template while the remaining seven samples are used for the verification process. To assess the performance of a biometric system, we can compute the Receiver Operating Characteristic (ROC) curve. This curve plots the False Match Rate (FMR) (*i.e*., accepted impostor attempts) on the x-axis against the corresponding False Non-Match Rate (FNMR) (*i.e*., rejected genuine attempts) on the y-axis plotted parametrically as a function of the decision threshold. An illustration of the ROC curve is presented in Figure 4. The area under the curve (hatched zone) should be as low as possible to minimize recognition errors. The associated measure is called AUC (Area Under the ROC Curve) and is often considered a global performance criterion. The AUC values obtained for different sizes of the reduced template are plotted to help the comparison of one selection algorithm with the others. We consider this value in this study to quantify the efficiency of a minutiae selection method.

### 3.5. Ground truth

Table 1 shows the AUC value for the three used datasets considering the initial template as a reference (with all minutiae). The performance of MCC is lower than expected. We believe that the association between the MINDTCT extractor with MCC is sub-optimal. We are not interested in maximizing the performance of user verification with the matching algorithms but to estimate the impact of template reduction methods on performance.

**Table 1:** AUC values for the three datasets with Bozorth, MCC, and the commercial matching algorithms.

	**Bozorth3**	**MCC**	**Commercial**
FVC2002DB2	14% ± .14	10% ± .28	0.04% ± .06
FVC2004DB1	11.1% ± .18	18.4% ± .17	3.77% ± .09
FVC2004DB2	11.1% ± .09	18.9% ± .12	3.68% ± .07

These AUC values are considered in this study as the ground truth, i.e., the best performance when having all minutiae. The application of a template reduction algorithm will decrease the performance. A good reduction method should minimize this decrease in performance, meaning having the lowest AUC value.

## 4. Experimental results

In this section, we present the experimental results we obtained given the protocol described in the previous section. We first analyze the computation time required for template reduction with all tested methods.

### 4.1. Computation time for minutiae selection

The processing time to generate a reduced template for each method is analyzed. All computations have been realized under Matlab running on a PC with an Intel Core I7 4-core processor with a frequency of 2.8GHz and 16 GB of RAM. Table 2 shows the average reduction times for each method considering all datasets. This time criterion is of importance to draw a trade-off between the performance and the computation time in order to formulate recommendations to select a biometric template reduction method for both commercial systems and uses in research. The most simple methods (Truncation, Random truncation, and Media Y) are very fast with a low impact of the maximal number of minutia to keep. Others are much more slower, especially the K-means and MRGA. These two last methods (without considering their performance) cannot be used in a verification context.

**Table 2:** Average computation time for all the minutiae sizes for each reduction method.

***N*_*max*_**	**30**	**34**	**38**	**42**	**46**	**50**
Truncation	7.9 ms	7.6 ms	7.4 ms	7.2 ms	7 ms	6.8 ms
Random truncation	20.7 ms	19.9 ms	18.9 ms	17.4 ms	16.5 ms	14.6 ms
Barycenter	476 ms	409 ms	376 ms	364 ms	301 ms	244 ms
Incremental Barycenter	5387 ms	4747 ms	3988 ms	3435 ms	2789 ms	2206 ms
K-means	24.3 s	24 s	22.8 s	20.5 s	18.3 s	15.5 s
Median Y	171 ms	164 ms	193 ms	186 ms	189 ms	196 ms
MRGA	38 min	35 min	28 min	20 min	18min	13 min

### 4.2. Performance analysis

We analyze the performance of template reduction methods for the three datasets and three matching algorithms. We expect to minimize the degradation of performance compared with the initial template (without any reduction).

#### 4.2.1. Impact of template reduction

**Figure 5:**
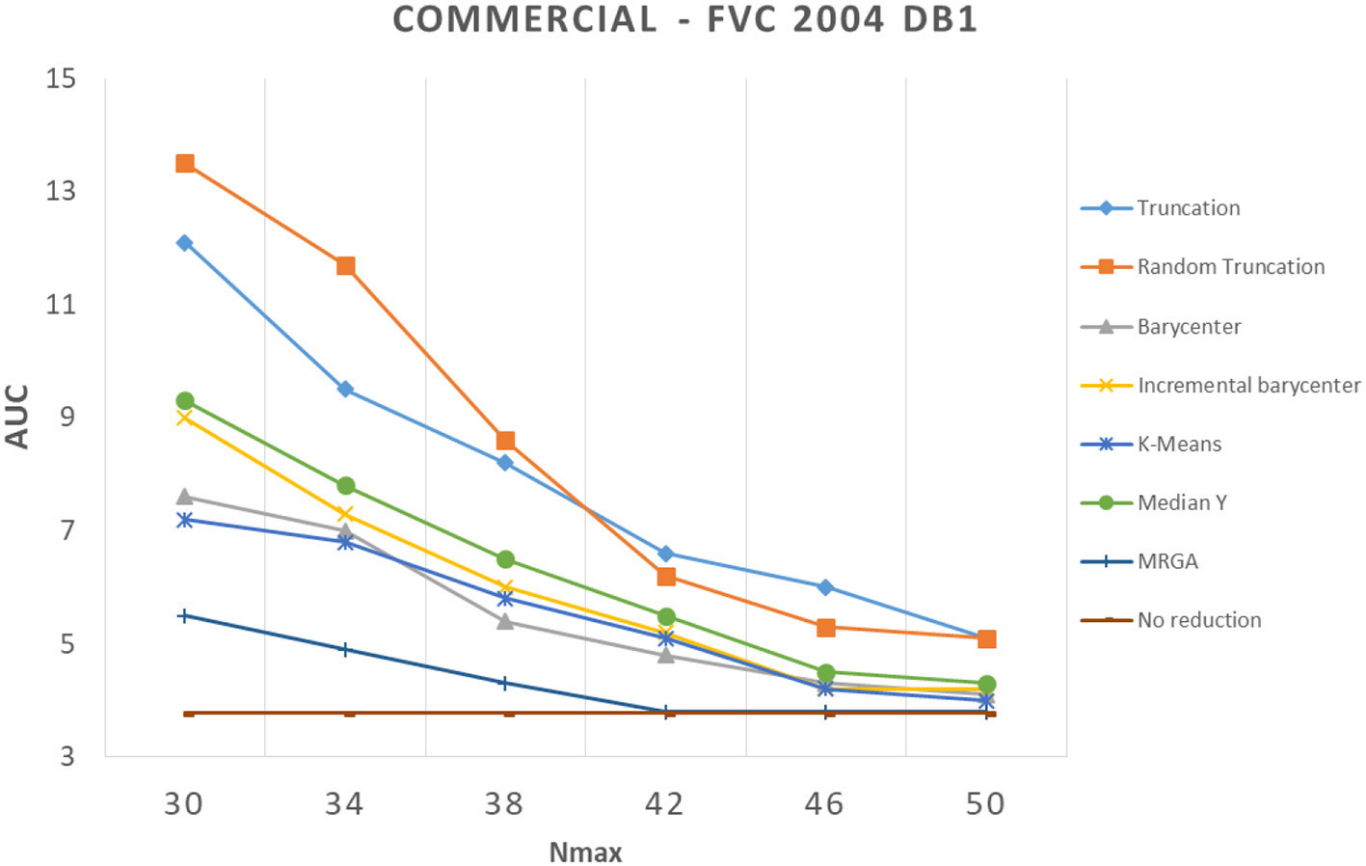
Comparison of template reduction methods on the FVC 2004 DB1 with the commercial matching algorithm.

**Figure 6:**
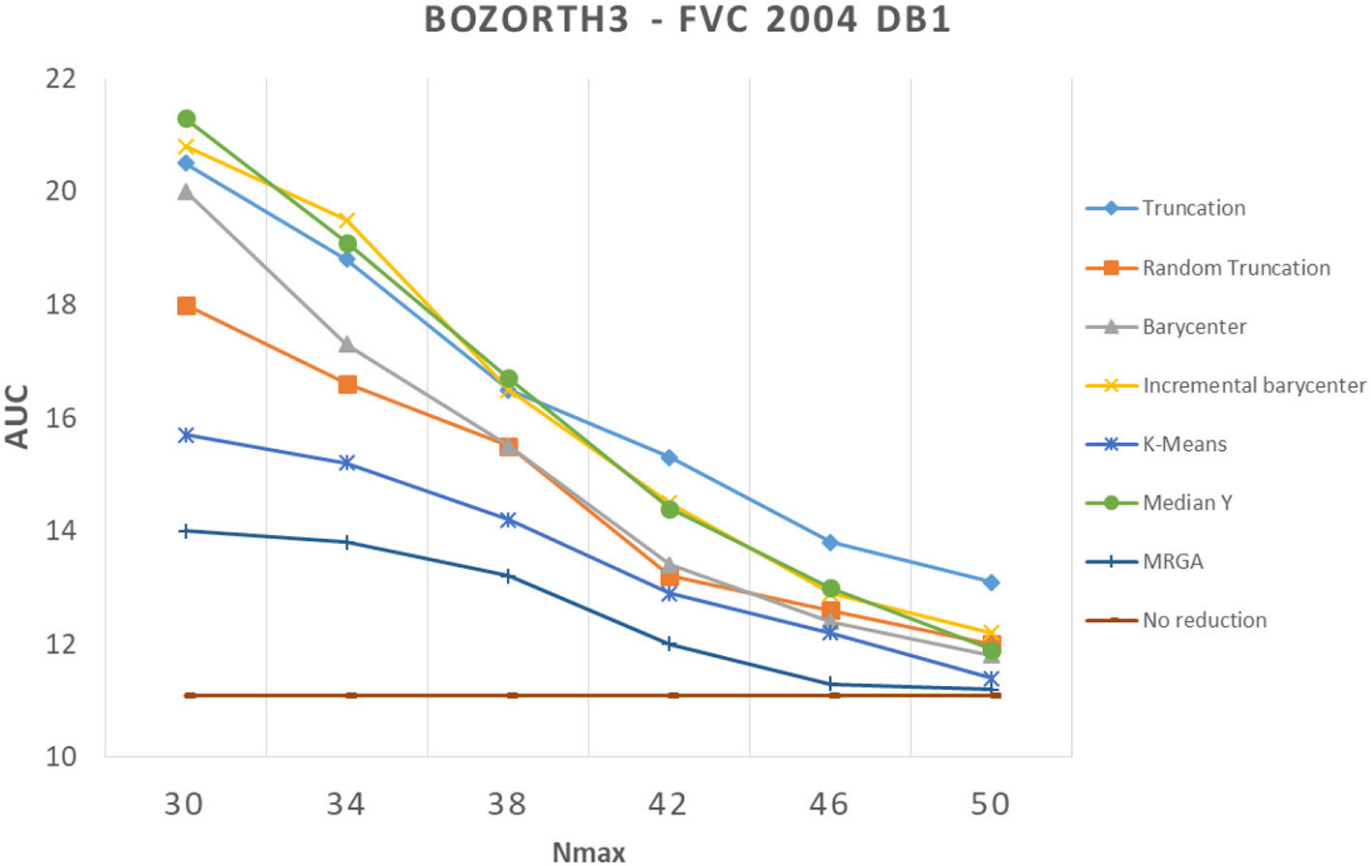
Comparison of template reduction methods on the FVC 2004 DB1 with the Bozorth3 matching algorithm.

**Figure 7:**
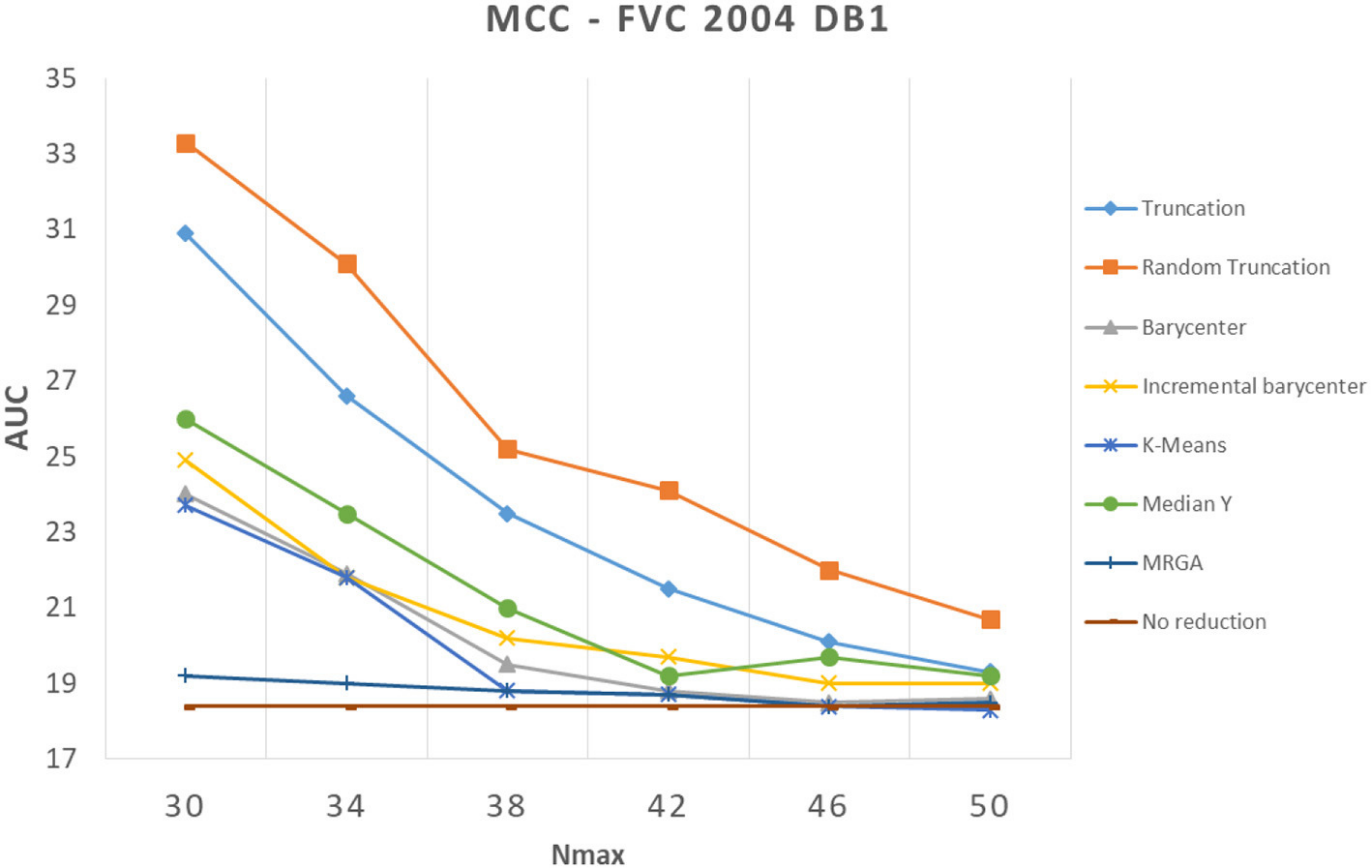
Comparison of template reduction methods on the FVC 2004 DB1 with the MCC matching algorithm.

In this section, we analyze how template reduction methods perform and their impacts on performance. Figures 5–7 present the evolution of the performance for each reduction template method with the three matching algorithms on the FVC 2004 DB1 dataset as illustration. As a reference, we considered the performance when using the initial template (no reduction). From these curves, We can draw the following conclusions.

First, we can see that the reduction of minutiae has the consequence of a decrease in performance. This is not a surprise to have this result, it was expected. For example, for the most efficient matching algorithm (commercial one), with *N*_*max*_ = 30 (where we keep only 30 minutiae from the template), we have an AUC value between 5.5% and 13.5% with template reduction methods, while using the initial template, we have an AUC value of 3.77% (see Table 1). The decrease in performance for each template reduction method is very different, we analyze it later.

Second, the relative template reduction methods have a very similar behavior for the MCC and commercial matching algorithms. This was expected, as these two algorithms are much more efficient even if Table 1 shows a better performance for Bozorth3. We focus more on our analysis on MCC and commercial matching algorithms.

We can observe that the reduction methods based on truncation are less efficient. The deterministic one is a bit more efficient than the random one, which suggests that the minutiae extractor adopts a sorting of minutiae with quality measures. The Median Y method has very different behaviors for the three matching algorithms, and this approach seems to be not very useful. Methods based on the computation of the barycenter (static or incremental computation) have similar behaviors and provide globally good results. The static version is much more efficient considering its computation time (see Table 2), this solution is a better choice.

Now, we consider the two slowest methods. The K-means method provides good results. The degradation of performance (estimated by the AUC value) for *N*_*max*_ = 30, as for example, is 7.2% instead of 3.7%, without any reduction for the commercial matching algorithm. Finally, the MRGA reduction method provides the best results for all the matching algorithms. As an illustration, the degradation of performance for *N*_*max*_ = 30 is 5.5% instead of 3.7%, without any reduction for the commercial matching algorithm. Of course, this method is not acceptable in a verification context, as its computation is too important (see Table 2).

#### 4.2.2. Reduction vs. performance

**Figure 8:**
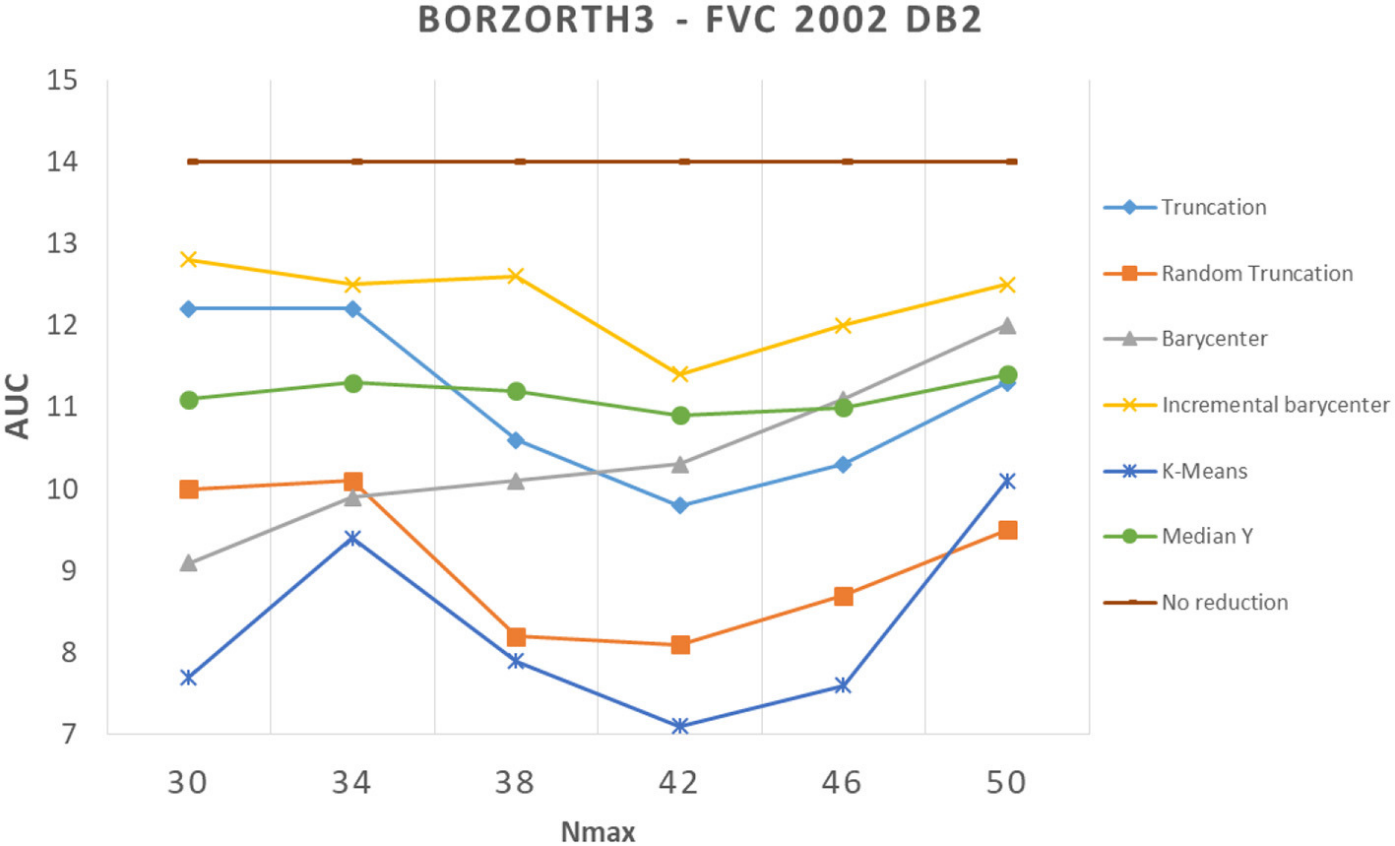
Comparison of template reduction methods on the FVC 2002 DB2 with the Bozorth3 matching algorithm.

We showed in the previous section that applying a reduction method usually decreases performance. In our experiments, we found a counter-example with the Bozorth3 matching algorithm on the FVC 2002 DB2 dataset (see Figure 8). We can see that all reduction methods improved greatly the performance. To better understand these results, we analyzed images from which minutiae templates are extracted. We observed that the images show capture artifacts (minutiae from a latent fingerprint), which mislead the minutiae extraction algorithm. It shows that the minutiae extractor detects false minutiae.

**Figure 9:**
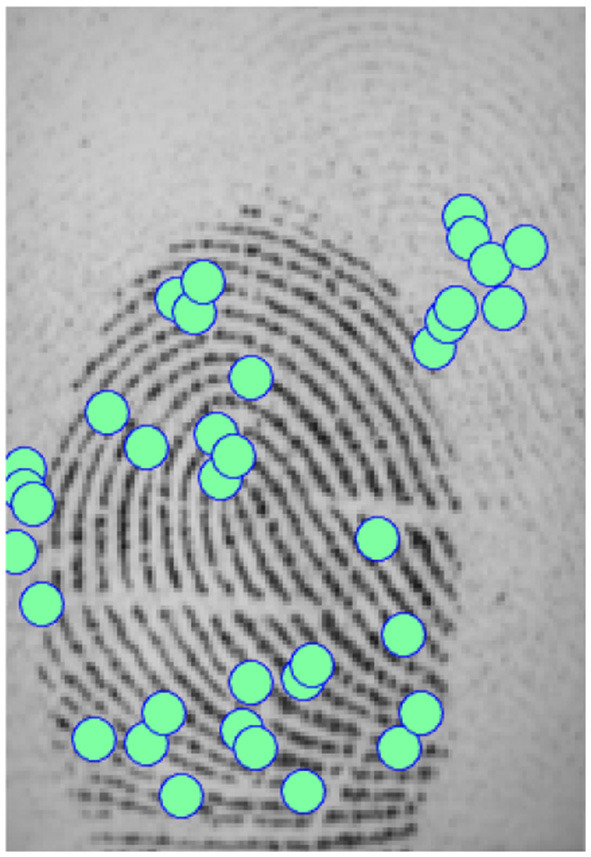
Example of fingerprint with minutiae artifacts.

Figure 9 illustrates this remark. We note that many minutiae are falsely detected due to capture artifacts. To validate our assumption, we took a sample of the FVC 2002 DB2 database, and we removed the minutiae from artifacts in the initial template (suppression of detected minutiae in the background of the fingerprint image). Then, we applied the different selection methods to these “cleaned" templates. We evaluated these new templates with the same methodology as before. We observed so-called “normal" performances (with a better performance than the initial template), that is, the reduction methods deteriorate the performance of the system compared with the initial template, as expected. This surprising result shows the benefit to suppress all minutiae in the background of the fingerprint as pre-processing of template reduction methods.

### 4.3. Discussion

Considering all results, we can draw many interesting conclusions. Of course, using a template reduction method permits to limit the data storage and the computation time for the matching process. This could be very important in embedded systems like smart cards having such constraints.

We also showed that suppressing spurious minutiae with a background detection is an interesting pre-processing before template reduction.

The different tested reduction methods have different behaviors in terms of performance and computation time. MRGA is the most efficient one, but it is very slow. The Barycenter method is a good compromise between performance and computation time. In an operational context, We propose to use the MRGA method during enrollment in order to have the best performance even if it is long as it is only conducted once. For user verification, the barycenter solution could be used, as it is fast and provides good results.

## 5. Conclusion and perspectives

This study has first presented the context of template reduction of digital fingerprints. We realized a literature review of reduction methods. All methods have been tested on three well-known biometric datasets often used in biometric competitions and on two academic comparison algorithms –Bozorth3 and MCC– and a commercial one. We have to mention that no code optimization was carried out in our study, and the proposed methods have been developed under Matlab; hence, we may hope to consequently reduce the computation time. We have shown that the best methods for reducing fingerprint templates are mostly those offering a good spatial distribution of the minutiae in the reduced template. We proposed to use the MRGA method for enrollment and the barycenter one for user verification for a real and efficient implementation.

From perspective, we believe that there is room for improvement of template reduction methods. We list in the following some possible strategies to define new methods.

The visual representation of the selection on the same template should give crucial lightening to find the best minutiae. However, there are too many differences between the selected minutiae of two different methods like K-means and MRGA to derive easily a new strategy of selection, an in-depth investigation remains to be conducted.

It may be noted that the performance of a reduction template method can be dependent on the matching algorithm even if MCC and the commercial one provided similar results. Hence, a method may be efficient for one and not the others, while another should be rather efficient for any matching algorithm. Defining a template reduction method using the matching algorithm as a core component could be proposed. We could, for example, select minutiae that have a high impact on the matching score between a reduced template and the initial one.

It might also be possible to consider works on minutiae quality to select the most interesting ones for reduction. We plan to work on this approach in future.

## Data availability statement

The original contributions presented in the study are included in the article/supplementary material, further inquiries can be directed to the corresponding author.

## Author contributions

BV and CR have contributed to the source code implementation and experiments. All authors have contributed to the concepts, methods, and authoring of the article. All authors contributed to the article and approved the submitted version.
